# Examinations

**Published:** 1868-12

**Authors:** 


### EXAMINATIONS.

On the 1st and 2d of December, the State Board of Dental Examiners held a session at Columbus, for the examination of those who might present themselves, that their qualifications to properly practice in the Dental profession might be testified to by that Board. A work of this kind is no easy matter—to bring out in clear view the measure of the professional attainments of those with whom the committee would necessarily be but slightly acquainted. The examination of the office or college student is a very easy affair, compared with the work of this committee. The preceptor, if he is faithful, knows the weak and the strong points of his pupil, and the pupil knows something, perhaps much, of the method of procedure of his preceptor.

In the examination of practitioners of experience, it is not proper or just to enter into minutiæ of details, but to ascertain to what extent general principles are possessed, upon which correct practice must be based.

The following persons having passed a very thorough and satisfactory examinatian, received the certificate from the Board to that effect, viz.: Drs. J. C. Hardy, of Cincinnati; Joseph Dunlap and French, of Chillicothe; I. Williams, of New Philadelphia; Scott, of Defiance; Talbert, of Galion, all being practitioners of from ten to twenty-five years.

It is exceedingly gratifying to know that our old and highly respectable practitioners desire to avail themselves of a public and legal recognition of their professional attainments and ability, and we have no hesitation in saying that the public may, with a large measure of confidence, rely upon the attainments and ability of those who bear with them a certificate of this Board.

The next regular session of this Board will be held the 1st of June next. A called meeting of the Board, however, will be held at any time, when a sufficient number will signify their desire to appear before the Board to warrant such a call.

The regular meetings of the Board will be held at Columbus. The called meetings may be held at any place in the vicinity of which five or more applicants may reside. A desire for such meetings of the Board, both in regard to time and place, may be made known to any of its members. T.

